# Supplementary material for: Characterization of Plasmodium falciparum NEDD8 and identification of cullins as its substrates
Source: Sci Rep. 2020 Nov 19;10:20220. doi: 10.1038/s41598-020-77001-5 (PMC7677368; doi:10.1038/s41598-020-77001-5)
Supplement: Supplementary file 5 — Supplementary Information. [file 41598_2020_77001_MOESM5_ESM.pdf]

Characterization of *Plasmodium falciparum* NEDD8 and identification of cullins as its substrates

Manish Bhattacharjee[1,#], Navin Adhikari[1,#], Renu Sudhakar[1], Zeba Rizvi[1], Divya Das[1], R Palanimurugan[1,2], Puran Singh Sijwali[1,2,*]

```
XP_001349866.1    ------------------------------------------------------------   0
HsNEDD8           ------------------------------------------------------------   0
XP_001350369.1    ------------------------------------------------------------   0
XP_001350526.1    MQIFVKTLTGKTITLDVEPSDTIENVKAKIQDKEGIPPDQQRLIFAGKQLEDGRTLSDYN   60

XP_001349866.1    ------------------------------------------------------------   0
HsNEDD8           ------------------------------------------------------------   0
XP_001350369.1    ------------------------------------------------------------   0
XP_001350526.1    IQKESTLHLVLRLRGGMQIFVKTLTGKTITLDVEPSDTIENVKAKIQDKEGIPPDQQRLI   120

XP_001349866.1    ------------------------------------------------------------   0
HsNEDD8           ------------------------------------------------------------   0
XP_001350369.1    ------------------------------------------------------------   0
XP_001350526.1    FAGKQLEDGRTLSDYNIQKESTLHLVLRLRGGMQIFVKTLTGKTITLDVEPSDTIENVKA   180

XP_001349866.1    ------------------------------------------------------------   0
HsNEDD8           ------------------------------------------------------------   0
XP_001350369.1    ------------------------------------------------------------   0
XP_001350526.1    KIQDKEGIPPDQQRLIFAGKQLEDGRTLSDYNIQKESTLHLVLRLRGGMQIFVKTLTGKT   240

XP_001349866.1    ------------------------------------------------------------   0
HsNEDD8           ------------------------------------------------------------   0
XP_001350369.1    ------------------------------------------------------------   0
XP_001350526.1    ITLDVEPSDTIENVKAKIQDKEGIPPDQQRLIFAGKQLEDGRTLSDYNIQKESTLHLVLR   300

                                                    3132
XP_001349866.1    ----MQILVKTLTGKRQSFNFEPSSSVFQIKMAIEEREGIDAKQIRLIYSGKQMHDDMKL   56
HsNEDD8           ----MLIKVKTLTGKEIEIDIEPTDKVERIKERVEEKEGIPPQQQRLIYSGKQMNDEKTA   56
XP_001350369.1    ----MQIFVKTLTGKTITLDVEPSDTIENVKAKIQDKEGIPPDQQRLIFAGKQLEDGRTL   56
XP_001350526.1    LRGGMQIFVKTLTGKTITLDVEPSDTIENVKAKIQDKEGIPPDQQRLIFAGKQLEDGRTL   360
                                                    **

                                 72
XP_001349866.1    SDYRVVPGSTVHMILQLRGG----------------------------------------   76
HsNEDD8           ADYKILGGSVLHLVLALRGGGGLRQ-----------------------------------   81
XP_001350369.1    SDYNIQKESTLHLVLRLRGGAIEPSLAQLAQKYNCQKLICRKCYARLHPRATNCRNKKCG   116
XP_001350526.1    SDYNIQKESTLHLVLRLRGGF---------------------------------------   381
                                 *

XP_001349866.1    ------------   76
HsNEDD8           ------------   81
XP_001350369.1    RTNQLRPKKKLK   128
XP_001350526.1    ------------   381
```

Fig. S1. Sequence alignment of the BLAST hits with human NEDD8. Shown is the sequence alignment of human NEDD8 (HsNEDD8) with the top 3 BLAST hits of the *P. falciparum* genome database (XP_001349866.1, XP_001350369.1 and XP_001350526.1). XP_001350526.1 is a polyubiquitin protein containing 5 ubiquitin domains; domains 1-4 are highlighted in different colours and the 5th domain is in blue font. XP_001350369.1 is a ubiquitin-ribosomal protein eL40; the ubiquitin domain is in blue font and the ribosomal protein sequence is in red font. The amino acid residues marked with asterisks differentiate ubiquitin and NEDD8 for being correctly recognized by their respective enzymatic machinery. Numbers on the top of the alignment indicate the position of that amino acid residue in human NEDD8 and XP_001349866.1. The filled arrow represents predicted cleavage sites to generate ubiquitin, and the open arrow represents processing site to remove the C-terminal tail in ubiquitin and NEDD8.

```
                                        10        20        30        40        50        60        70        80        90
                                         |         |         |         |         |         |         |         |         |
C.depauperatus(A0A1E3JCZ1)    ---MIVKVKTLTGKEVDI-DVQPDMTIGKVKEKVEEKAGIPPVQQRLISGGKAMNDEKTIQDYKIKAAD--VIHLVLALRGGRA-------------
K.bestiolae(A0A1B9GAI6)       ---MIVKVKTLTGKEVDI-DVQPDMTINKVKERVEEKAGIPPVQQRLIFGGKAMADDKAISDYKINAGA--VIHLVLALRGGR--------------
K.mangroviensis(A0A1B9IIT2)   ---MIVKVKTLTGKEVDI-DVQPDMTINKVKERVEEKAGIPPVQQRLIFGGKAMADDKAIQDYKINAGA--VIHLVLALRGGR--------------
C.gattii(A0A095CBA7)          ---MIVKVKTLTGKEVDI-DVQPDMTISKVKERVEEKAGIPPVQQRLIFGGKAMGDDKTIQDYKIQAGA--AIHLVLALRGGRA-------------
C.neoformans(J9VQ81)          ---MIVKVKTLTGKEVDI-DVQPDMTISKVKERVEEKAGIPPVQQRLIFGGKAMGDDKTIQDYKIQAGA--AIHLVLALRGGRA-------------
T.mesenterica(A0A4V1M4Z3)     ---MLIKVKTLTGKEIEL-DVQPETKVSKVKENVEEKAGIPPIQQRLIFGGKAMNDEKELGFYGVPAGG--VIHLVLALRGGR--------------
L.edodes(A0A1Q3EGM6)          ---MLIKVRTLTGKELEI-DVDSDAKIFTIKEKVEEQQGIPPVQQRLIFGGKQLDDDKHITETNIVAGS--TLHLVLALRGGR--------------
A.parvum(A0A023FVX4)          ---MLIKVKTLTGKEIEI-DIEPTDKVERIKERVEEKEGIPPAQQRLIFSGKQMNDDKTAADYKVTGGS--VLHLVLALRGG-EWCACYL-------
A.americanum(B5M745)          ---MLIKVKTLTGKEIEI-DIEPTDKVERIKERVEEKEGIPPAQQRLIFSGKQMNDDKTAADYKVTGGS--VLHLVLALRGG-EWRACNQ-------
H.vulgaris(T2MDB3)            ---MLIKVKTLTGKEIEI-DIEPNDKVERIKERVEEKEGIPPAQQRLIFSGKQMNDDKTAQDYKVSGGS--VLHLVLALRGG---------------
A.ventricosus(A0A4Y2GFW1)     ---MLIKVKTLTGKEIEI-DIEPTDKVERIKERVEEKEGIPPAQQRLIFSGKQMNDDKTAFDYKVTGGS--VLHLVLALRGGYPSESCFC-------
A.cerana(A0A2A3E9N1)          ---MLIKVKTLTGKEIEI-DIEPTDKVERIKERVEEKEGIPPQQQRLIFSGKQMNDEKTAQDYKVQGGS--VLHLVLALRGGL--------------
A.planipennis(A0A1W4WES4)     ---MLIKVKTLTGKEIEI-DIEPTDKVERIKERVEEKEGIPPQQQRLIFSGKQMNDEKTAQDYKVQGGS--VLHLVLALRGGLQ-------------
T.castaneum(D6WL56)           ---MLIKVKTLTGKEIEI-DIEPTDKVERIKERVEEKEGIPPQQQRLIFSGKQMNDEKTAQDYKVQGGS--VLHLVLALRGGTSF------------
P.humanus(E0VU21)             ---MLIKVKTLTGKEIEI-DIEPTDKVERIKERVEEKEGIPPQQQRLIFSGKQMNDEKTAQDYKVQGGS--VLHLVLALRGGYSNIISAIN------
R.pedestris(R4WQJ5)           ---MLIKVKTLTGKEIEI-DIEPTDKVERIKERVEEKEGIPPQQQRLIFSGKQMNDEKTAADYKVQGGS--VLHLVLALRGGHNVSH----------
P.albipes(T1DFZ4)             ---MLIKVKTLTGKEIEI-DIEPTDKVDRIKERVEEKEGIPPQQQRLIFSGKQMNDDKTAQDYKVQGGS--VLHLVLALRGGHQ-------------
A.gambiae(Q7Q3J6)             ---MLIKVKTLTGKEIEI-DIEPTDKVDRIKERVEEKEGIPPQQQRLIFSGKQMNDDKTAQDYKVQGGS--VLHLVLALRGGLLL------------
A.albopictus(A0A023EDB2)      ---MLIKVKTLTGKEIEI-DIEPTDKVDRIKERVEEKEGIPPQQQRLIFSGKQMNDDKTAQDYKVQGGS--VLHLVLALRGGRL-------------
D.melanogaster(X2J935)        ---MLIKVKTLTGKEIEI-DIEPTDKVDRIKERVEEKEGIPPQQQRLIFSGKQMNDDKTAADYKVQGGS--VLHLVLALRGGDSILTPCV-------
D.citri(A0A1S3DU76)           ---MLIKVKTLTGKEIEI-DIEPTDKVERIKERVEEKEGIPPQQQRLIFSGKQMNDEKTAAEYKVQGGS--VLHLVLALRGGHSVIQSSLDQVLL--
R.appendiculatus(A0A131Z921)  ---MLIKVKTLTGKEIEI-DIEPNDKVERIKERVEEKEGIPPAQQRLIYSGKQMNDEKTATEYKVQGGS--VLHLVLALRGGQWR------------
L.unguis(A0A1S3KDM3)          ---MLIKVKTLTGKEIEI-DIEPTDKVERIKERVEEKEGIPPQQQRLIFSGKQMNDDKTAADYKVQGGS--VLHLVLALRGGHRGH-----------
S.pistillata(A0A2B4RW20)      ---MLIKVKTLTGKEIEI-DIEPTDKVERIKERVEEKEGIPPPQQRLIFSGKQMNDDKTAADYKIQGGS--VLHLVLALRGGLF-------------
C.horridus(A0A0K8RYZ5)        ---MLIKVKTLTGKEIEI-DIEPTDKVERIKERVEEKEGIPPQQQRLIYSGKQMNDEKTAADYKIQGGS--VLHLVLALRGGLR-------------
O.mykiss(C1BFL8)              ---MLIKVKTLTGKEIEI-DIEPTDKVERIKERVEEKEGIPPQQQRLIYSGKQMNDEKTAADYKIQGGS--VLHLVLALRGGLVCHCNRIQLIV---
S.salar(B5X8K6)               ---MLIKVKTLTGKEIEI-DIEPTDKVERIKERVEEKEGIPPQQQRLIYSGKQMNDEKTAADYKIQGGS--VLHLVLALRGGEVLHCPTSLLLAL--
G.aculeatus(G3PMC3)           ---MLIKVKTLTGKEIEI-DIEPTDKVERIKERVEEKEGIPPQQQRLIYSGKQMNDEKTAADYKIQGGS--VLHLVLALRGGSRPRR----------
M.mola(A0A3Q4BER3)            ---MLIKVKTLTGKEIEI-DIEPTDKVERIKERVEEKEGIPPQQQRLIYSGKQMNDEKTAADYKIQGGS--VLHLVLALRGGSTLRRPCTRLSPTS-
X.couchianus(A0A3B5LLA6)      ---MLIKVKTLTGKEIEI-DIEPTDKVERIKERVEEKEGIPPQQQRLIYSGKQ-NDEKTAADYKIQGGS--VLHLVLALRGGSEPH-----------
P.reticulata(A0A3P9QEH4)      ---MLIKVKTLTGKEIEI-DIEPTDKVERIKERVEEKEGIPPQQQRLIYSGKQMNDEKTAADYKIQGGS--VLHLVLALRGGSEPHRSCTRLSPSS-
P.mexicana(A0A3B3YIE0)        ---MLIKVKTLTGKEIEI-DIEPTDKVERIKERVEEKEGIPPQQQRLIYSGKQMNDEKTAADYKIQGGS--VLHLVLALRGGSEPHRSCTRLSPSS-
P.latipinna(A0A3B3UNR2)       ---MLIKVKTLTGKEIEI-DIEPTDKVERIKERVEEKEGIPPQQQRLIYSGKQMNDEKTAADYKIQGGS--VLHLVLALRGGSEPHRSCTRLSPSS-
T.nigroviridis(H3CR82)        ---MLIKVKTLTGKEIEI-DIEPTDKVERIKERVEEKEGIPPQQQRLIYSGKQMNDEKTAADYKIQGGS--VLHLVLALRGGSPS------------
A.limnaeus(A0A2I4AWV4         ---MLIKVKTLTGKEIEI-DIEPTDKVERIKERVEEKEGIPPQQQRLIYSGKQMNDEKTAADYKIQGGS--VLHLVLALRGGSPPRSCSTHLSSSS-
A.percula(A0A3P8TSK8)         ---MLIKVKTLTGKEIEI-DIEPTDKVERIKERVEEKEGIPPQQQRLIYSGKQMNDEKTAADYKIQGGS--VLHLVLALRGGSELHSRCIHVSSSS-
L.chalumnae(H3A9S3)           ---TIAKVQTLTGKEIEI-DIEPTDKVERIKERVEEKEGIPPQQQRLIYSGKQMNDEKTAADYKIQGGS--VLHLVLALRGGSR-------------
E.kenyoni(A0A2Y9IJM8)         ---MLIKVKTLTGKEIEI-DIEPTDKVERIKERVEEKEGIPPQQQRLIYSGKQMNDEKTAADYKILGGS--VLHLVLALRGGGGLRQ----------
F.catus(M3W4C3)               ---MLIKVKTLTGKEIEI-DIEPTDKVERIKERVEEKEGIPPQQQRLIYSGKQMNDEKTAADYKILGGS--VLHLVLALRGGGGLRQ----------
C.lupus(F1P890)               ---MLIKVKTLTGKEIEI-DIEPTDKVERIKERVEEKEGIPPQQQRLIYSGKQMNDEKTAADYKILGGS--VLHLVLALRGGGGLRQ----------
E.europaeus(A0A1S3AKE6)       ---MLIKVKTLTGKEIEI-DIEPTDKVERIKERVEEKEGIPPQQQRLIYSGKQMNDEKTAADYKILGGS--VLHLVLALRGGGGLRQ----------
E.caballus(A0A3Q2HYF4)        ---MLIKVKTLTGKEIEI-DIEPTDKVERIKERVEEKEGIPPQQQRLIYSGKQMNDEKTAADYKILGGS--VLHLVLALRGGGGLRQ----------
```

```
C.porcellus(A0A286XT26)            ---MLIKVKTLTGKEIEI-DIEPTDKVERIKERVEEKEGIPPQQQRLIYSGKQMNDEKTAADYKILGGS--VLHLVLALRGGGGLRQ----------
P.coquereli(A0A2K6F9I7)            ---MLIKVKTLTGKEIEI-DIEPTDKVERIKERVEEKEGIPPQQQRLIYSGKQMNDEKTAADYKILGGS--VLHLVLALRGGGGLRQ----------
C.angolensis(A0A2K5IA72)           ---MLIKVKTLTGKEIEI-DIEPTDKVERIKERVEEKEGIPPQQQRLIYSGKQMNDEKTAADYKILGGS--VLHLVLALRGGGGLRQ----------
U.maritimus(A0A384DJ45)            ---MLIKVKTLTGKEIEI-DIEPTDKVERIKERVEEKEGIPPQQQRLIYSGKQMNDEKTAADYKILGGS--VLHLVLALRGGGGLRQ----------
P.macrocephalus(A0A2Y9FV62)        ---MLIKVKTLTGKEIEI-DIEPTDKVERIKERVEEKEGIPPQQQRLIYSGKQMNDEKTAADYKILGGS--VLHLVLALRGGGGLRQ----------
O.divergens(A0A2U3W261)            ---MLIKVKTLTGKEIEI-DIEPTDKVERIKERVEEKEGIPPQQQRLIYSGKQMNDEKTAADYKILGGS--VLHLVLALRGGGGLRQ----------
U.horribilis(A0A3Q7VHA4)           ---MLIKVKTLTGKEIEI-DIEPTDKVERIKERVEEKEGIPPQQQRLIYSGKQMNDEKTAADYKILGGS--VLHLVLALRGGGGLRQ----------
V.vulpes(A0A3Q7TQ17)               ---MLIKVKTLTGKEIEI-DIEPTDKVERIKERVEEKEGIPPQQQRLIYSGKQMNDEKTAADYKILGGS--VLHLVLALRGGGGLRQ----------
C.ursinus(A0A3Q7NXY2)              ---MLIKVKTLTGKEIEI-DIEPTDKVERIKERVEEKEGIPPQQQRLIYSGKQMNDEKTAADYKILGGS--VLHLVLALRGGGGLRQ----------
N.schauinslandi(A0A2Y9HNT6)        ---MLIKVKTLTGKEIEI-DIEPTDKVERIKERVEEKEGIPPQQQRLIYSGKQMNDEKTAADYKILGGS--VLHLVLALRGGGGLRQ----------
N.asiaeorientalis(A0A341C3R4)      ---MLIKVKTLTGKEIEI-DIEPTDKVERIKERVEEKEGIPPQQQRLIYSGKQMNDEKTAADYKILGGS--VLHLVLALRGGGGLRQ----------
H.sapiens(Q15843)                  ---MLIKVKTLTGKEIEI-DIEPTDKVERIKERVEEKEGIPPQQQRLIYSGKQMNDEKTAADYKILGGS--VLHLVLALRGGGGLRQ----------
L.pardinus(A0A485MAU2)             ---MLIKVKTLTGKEIEI-DIEPTDKVERIKERVEEKEGIPPQQQRLIYSGKQMNDEKTAADYKILGGS--VLHLVLALRGGGGFRQ----------
N.vison(U6CT53)                    ---MLIKVKTLTGKEIEI-DIEPTDKVERIKERVEEKEGIPPQQQRLIYSGKQMNDEKTAADYKILGGS--VLHLVLALRGGGDLRQ----------
P.abelii(H2NKV7)                   ---MLIKVKTLTGKEIEI-DIEPTDKVERIKERVEEKEGISPQQQRLIYSGKQMNDEKTAADYKILGGS--VLHLVLALRGGGGLRQ----------
M.auratus(A0A1U8CYD2)              ---MLIKVKTLTGKEIEI-DIEPTDKVERIKERVEEKEGIPPQQQRLIYSGKQMNDEKTAADYKILGGS--VLHLVLALRGGGGLGQ----------
C.griseus(G3HDD4)                  ---MLIKVKTLTGKEIEI-DIEPTDKVERIKERVEEKEGIPPQQQRLIYSGKQMNDEKTAADYKILGGS--VLHLVLALRGGGGLGQ----------
I.tridecemlineatus(qI3N6M7)        ---MLIKVKTLTGKEIEI-DIEPTDKVERIKERVEEKEGIPPQQQRLIYSGKQMNDEKTAADYKILGGS--VLHLVLALRGGDSLRQ----------
G.gorilla(G3RJ38)                  ---------TLTGKEIEI-DIEPTDKVERIKERVEEKEGIPPQQQRLIYSGKQMNDEKTAADYKILGGS--VLHLVLALRGGGGLRQ----------
A.melanoleuca(D2HG47)              --------QTLTGKEIEI-DIEPTDKVERIKERVEEKEGIPPQQQRLIYSGKQMNDEKTAADYKILGGS--VLHLVLALRGGGGLRQ----------
O.aries(W5QCY9)                    -------FQTLTGKEIEI-DIEPTDKVERIKERVEEKEGIPPQQQRLIYSGKQMNDEKTAADYKILGGS--VLHLVLALRGGGGLRQ----------
U.americanus(A0A452SHB5)           ---NPCLFQTLTGKEIEI-DIEPTDKVERIKERVEEKEGIPPQQQRLIYSGKQMNDEKTAADYKILGGS--VLHLVLALRGGGGLRQ----------
A.mississippiensis(A0A151M8R3)     ---MLIKVKTLTGKEIEI-DIEPTDKVERIKERVEEKEGIPPQQQRLIYSGKQMNDEKTAADYKIQGGS--VLHLVLALRGGGLR------------
G.gallus(A0A1D5PRI6)               ---MLIKVKTLTGKEIEI-DIEPTDKVERIKERVEEKEGIPPQQQRLIYSGKQMNDEKTAADYKIQGGS--VLHLVLALRGGGGVKQWGRF------
C.milii(V9LK01)                    ---MLIKVKTLTGKEIEI-DIEPTDKVERIKERVEEKEGIPPQQQRLIYSGKQMNDEKTASDYKIQGGS--VLHLVLALRGGAGL------------
M.domestica(F6T1M0)                ---MLIKVKTLTGKEIEI-DIEPTDKVERIKERVEEKEGIPPQQQRLIYSGKQMNDEKTAADYKIQGGS--VLHLVLALRGGGGLGR----------
R.norvegicus(A0A0G2K4G3)           ---MVLEQKTLTGKEIEI-DIEPTDKVERIKERVEEKEGIPPQQQRLIYSGKQMNDEKTAADYKILGGS--VLHLVLALRGGGGLGQ----------
L.africana(G3U8Y0)                 ---MFIFQKTLTGKEIEI-DIEPTDKVERIKERVEEKEGIPPQQQRLIYSGKQMNDEKTAADYKILGGS--VLHLVLALRGGGGIRK----------
M.zebra(A0A3P9D203)                ---MLIKVKTLTGKEIEI-DIEPTDKVERIKERVEEKEGIPPQQQRLIYSGKQMNDEKTAADYKIQGGS--VLHLVLALRGGCLLHHRTTLCSTPAV
S.dumerili(A0A3B4TTJ4)             ---MLIKVKTLTGKEIEI-DIEPTDKVERIKERVEEKEGIPPQQQRLIYSGKQMNDEKTAADYKIQGGS--VLHLVLALRGGQVLHSPRCLYSKPAL
H.comes(A0A3Q2Y7I5)                ---MLIKVKTLTGKEIEI-DIEPTDKVERIKERVEEKEGIPPQQQRLIYSGKQMNDEKTAADYKIQGGS--VLHLVLALRGGKAHHTPDVQLASS--
D.rerio(F1QMF9)                    ---MLIKVKTLTGKEIEI-DIEPTDKVERIKERVEEKEGIPPQQQRLIYSGKQMNDEKTAADYKIQGGS--VLHLVLALRGGQIHQKPDRLLLNM--
A.mexicanus(A0A3B1JL70)            ---MLIKVKTLTGKEIEI-DIEPTDKVERIKERVEEKEGIPPQQQRLIYSGKQMNDEKTASDYKIQGGS--VLHLVLALRGGRIIY-----------
I.punctatus(E3TDT0)                ---MLIKVKTLTGKEIEI-DIEPTDKVERIKERVEEKEGIPPQQQRLIYSGKQMNDEKTAADYKIQGGS--VLHPVLALRGGRVHQHPNNLLQAL--
P.nattereri(A0A3B4EDC6)            ---MLIKVKTLTGKEIEI-DIEPTDKVERIKERVEEKEGIPPQQQRLIYSGKQMNDEKTAADYKIQGGS--VLHLVLALRGGREHQHPRNHLQAL--
E.lucius(C1BY35)                   ---MLIKVKTLTGKEIEI-DIEPTDKVERIKERVEEKEGIPPQQQRLIYSGKQMNDEETAADYKIQGGS--VLHLVLAPRGGLLYHCPSIQIHS---
M.albus(A0A3Q3QM89)                ---MLETFATLTGKEIEI-DIEPTDKVERIKERVEEKEGIPPQQQRLIYSGKQMNDEKTAADYKIQGGS--VLHLVLALRGGSVLHGPCCIFFSLS-
L.loa(A0A1I7W0I3)                  ---MLIKVKTLTGKEIEL-DIEPNDKVERIKEKVEEKEGIPPPQQRLIFAGKQMNDEKTAQDYKVAGGS--VLHLVLALRGGA--------------
A.grahami(A0A3N0XJQ5)              ---MHLFLKTLTGKEIEI-DIEPTDKVERIKERVEEKEGIPPQQQRLIYSGKQMNDEKTAADYKIQGGS--VLHLVLALRGGRLHQHLRSLLLTM--
L.corymbifera(A0A068SBG6)          ---MLIKVKTLTGKEIDI-DIEPTDKISRIKERVEEKEGIPPQQQRLIFSGKQMADEMTANEYSIVGGS--VLHLVLALRGGN--------------
R.microsporus(A0A0A1N8D5)          ---MLVKVKTLTGKEIEI-DIEPSDKISRIKERVEEKEGIPPQQQRLIYSGKQMSDEMTASQYGIEGGS--VLHLVLALRGGQ--------------
C.sinensis(H2KVL7)                 ---MLIKVKTLTGKEIEI-DIEPTDKVERIKERIEEKEGIPPPQQRLIFSGKQMHDEKVVSDYKIQGGS--VIHLVLSLRGGSR-------------
E.multilocularis(A0A068YBH6)       ---MLIKVKTLVGKEIEI-DIDQTDKIERIKERIEEKEGIPPPQQRLIFSGKQMNDEKTVNDYKIQGGS--VLHLVLALRGGQKH------------
D.discoideum(Q54XV3)               ---MLIKVKTLTGKEIEI-DIDPTDKIQRIKERVEEKEGIPPSQQRLIFGGKQMGDDKPASEYSIEGGS--VLHLVLALRGGL--------------
```

```
S.reilianum(E6ZVZ6)            ---MLIKVKTLTGKEIEL-DIEQTDKIQRIKERVEEKEGIPPAQQRLIFGGKQMHDEKTAKDFGVEGGS--VLHLVLALRGGSL-------------
A.ingoldii(A0A316YTF5)         ---MLIKVKTLTGKEIEL-DIEPTDKITRIKERVEEKEGIPPAQQRLIFGGKQMHDEKTAKEFGVEGGS--VLHLVLALRGGAFVGAMRG-------
A.amnicola(A0A1D1YNW4)         ---TMIKVKTLTGKEIEI-DIEPTDTIDRIKERVEEKEGIPPIQQRLIYAGKQLADDKTAKEYNIEGGS--VLHLVLALRGGLLS------------
M.truncatula(G7K8J5)           ---TMIKVKTLTGKEIEI-DIEPTDTIDRIKERVEEKEGIPPVQQRLIYAGKQLADDKTAKEYNIEGGS--VLHLVLALRGGN--------------
G.soja(A0A445GYG4)             ---TMIKVKTLTGKEIEI-DIEPTDTIDRIKERVEEKEGIPPVQQRLIYAGKQLADDKTAKEYNIEGGS--VLHLVLALRGGTY-------------
V.radiata(A0A1S3U8Q0)          ---TMIKVKTLTGKEIEI-DIEPTDTIDRIKERVEEKEGIPPVQQRLIYAGKQLADDKTAKEYNIEGGS--VLHLVLALRGGIY-------------
D.antarctica(P0C073)           ---TMIKVKTLTGKEIEI-DIEPTDTIDRVKERVEEKEGIPPVQQRLIYAGKQLADDKTAKDYNIEGGS--VLHLVLALRGGY--------------
T.urartu(M7ZY92)               ---TMIKVKTLTGKEIEI-DIEPTDTIDRIKERVEEKEGIPPVQQRLIYAGKQLADDKTAKDYNIEGGS--VLHLVLALRGGY--------------
P.dactylifera(A0A2H3X0H4)      ---TMIKVKTLTGKEIEI-DIEPTDTIDRIKERVEEKEGIPPVQQRLIYAGKQLADDKTAKDYNIEGGS--VLHLVLALRGGL--------------
B.rapa(V5M3G8)                 ---TMIKVKTLTGKEIEI-DIEPTDTIDRIKERVEEKEGIPPVQQRLIYAGKQLADDKTAKDYNIEGGS--VLHLVLALRGGFGLL-----------
S.lycopersicum(A0A3Q7I9N5)     ---TMIKVKTLTGKEIEI-DIEPTDTIDRIKERVEEKEGIPPVQQRLIYAGKQLADDKTAKDYNIEGGS--VLHLVLALRGGRL-------------
N.sylvestris(A0A1U7YZI3)       ---TMIKVKTLTGKEIEI-DIEPNDTIDRIKERVEEKEGIPPVQQRLIYAGKQLADDKTAKDYNIEGGS--VLHLVLALRGGRL-------------
S.splendens(A0A4D8ZGG0)        ---TMIKVKTLTGKEIEI-DIEPTDTIDRIKERVEEKEGIPPVQQRLIYAGKQLGDDKTAKDYNIEGGS--VLHLVLALRGGSH-------------
J.regia(A0A2I4E887)            ---TMIKVKTLTGKEIEI-DIEPTDTIDRIKERVEEKEGIPPVQQRLIYAGKQLGDDKTAKDYNIEGGS--VLHLVLALRGGSI-------------
C.melo(A0A1S3C469)             ---TMIKVKTLTGKEIEI-DIEPTDTIDRIKERVEEKEGIPPVQQRLIYAGKQLGDDKTAKDYNIEGGS--VLHLVLALRGGHL-------------
G.arboreum(A0A0B0N165)         ---TMIKVKTLTGKEIEI-DIEPTDTIDRIKERVEEKEGIPPVQQRLIYAGKQLADDKTARDYNIEGGS--VLHLVLALRGGCL-------------
N.nucifera(A0A1U7ZNS2)         ---TMIKVKTLTGKEIEI-DIEPTDTIERIKERVEEKEGIPPVQQRLIYAGKQLADDKTARDYNIEGGS--VLHLVLALRGGSL-------------
Z.marina(A0A0K9P544)           ---TMIKVKTLTGKEIEI-DIEPNDTIERIKERVEEKEGIPPVQQRLIYAGKQLSDDKTAKDYNIEGGS--VLHLVLALRGGGN-------------
A.macrogynus(A0A0L0S2I6)       ---MQIKVKTLTGKEIEI-DVEIDDKLTRVKEKVEEKEGIPPAQQRLIFGGKQMSDEKTLADYNIQGGS--VLHLVLSLRGGHC-------------
B.mutus(L8HUC3)                ---MLIKVKTLTRKEIEI-DTEPTDKVEQIKERVKEKEGIPPEQQRLTYSGRQINVKKTSADYKLLSGS--VLHLVLALRGGSDRPCSILPPCP---
B.prasinos(K8F2I6)             ---MMIKVKTLTGKEVEL-DVEVSDTIVRIKERMEEKEGIPPIQQRLIFGGKQMHDEKTAKDYNIEGGS--VIHMVLALRGGGCV------------
C.lustricola(A0A2T3AIS2)       ---MQIKVRTLTGKEIEL-DIEGNYLISQIKEQVEEKEGIPPVQQRLIYQGKQMADEKKASEYDLQGGN--VLHLVLALRGGQ--------------
G.clavigera(F0XRI8)            ---MLIKVRTLTGKEIEL-DIDSDYKVSQIKEKVEEKEGIPPVQQRLIYGGKQMVDDKTASDYALEGGA--TLHLVLALRGGL--------------
M.bolleyi(A0A136IM41)          ---MLIKVRTLTGKEIEL-DIEADYKVSQIKEKVEEKEGIPPVQQRLIYGGKQMVDDKSAADYALEGGA--TLHLVLALRGGSQ-------------
C.orbiculare(N4V966)           ---MLIKVRTLTGKEIEL-DIENEYKVSQIKEKVEEKEGIPPVQQRLIYGGKQMVDDKSASEYALEAGA--TLHLVLALRGGCSPDGF---------
C.fructicola(L2G896)           ---MLIKVRTLTGKEIEL-DIENEYKVSQIKEKVEEKEGIPPVQQRLIYGGKQMVDDKSASEYGLEAGA--TLHLVLALRGGKASDVC---------
C.adophora(A0A2V1C054)         ---MLIKVRTLTGKEIEL-DIEPDYKVSQIKEKVEEKEGIPPVQQRLIFGGKQMADEKTATEYALEGGA--TLHLVLALRGGVCL------------
U.pustulata(A0A1W5CZ69)        ---MNIKVRTLTGKEIEL-DIEPDYKVARIKERVEEKEGIPPVQQRLIYGGKQMADDKTAAEYSLEGGA--TLHLVLALRGGR--------------
L.maculans(E4ZWL8)             ---MQIKVRTLTGKEIEL-DIEADYKVSRIKERVEEKEGIPPAQQRLIYGGKQMADDKTAADYQLEGGA--TLHLVLALRGGC--------------
A.calidoustus(A0A0U5FUQ5)      ---MLIKVRTLTGKEIEL-DIEADYKVSRIKERVEEKEGIPPVQQRLIFGGKQMADDKTAQDYNLEGGA--TLHLVLALRGGCDAL-----------
G.moniliformis(W7LQF3)         ---MLIKVRTLTGKEIEL-DIESDYKVSQIKEKVEEKEGIPPVQQRLIHGGKQMTDDKTAAEYNLSAGD--TLHLVLALRGGRWMA-----------
F.oxysporum(W9II10)            ---MLIKVRTLTGKEIEL-DIESDYKVSQIKEKVEEKEGIPPVQQRLIHGGKQMTDDKTAAEYNLSAGD--TLHLVLALRGGRWMA-----------
T.guizhouense(A0A1T3CLM4)      ---MLIKVRTLTGKEIEL-DIESDYKVSQIKEKVEEKEGIPPVQQRLIHGGKQMTDDKTAAEYNLVAGD--TLHLVLALRGGRHFQ-----------
T.spiralis(A0A0V1BQN1)         MFKMLVKVKTLTGKEVEL-DIDASDLVERIKEKIEEKEGIPPAQQRLIHAGKQMSEGKTAAEYKITGGA--VLHLVLALRGGSL-------------
T.zimbabwensis(A0A0V1I5L9)     MLNMLVKVKTLTGKEVEL-DIDASDLVERIKEKIEEKEGIPPAQQRLIHAGKQMSEGKTAAEYKITGGA--VLHLVLALRGGSL-------------
T.richinella(A0A0V1BQN1)       ---MLVKVKTLTGKEVEL-DIDASDLVERIKEKIEEKEGIPPAQQRLIHAGKQMSEGKTAAEYKITGGAKTMAEFVLNLGYGSDDVL----------
A.thaliana(O65381)             ---MEIKVKTLTEKQIDI-EIELTDTIERIKERIEEKEGIPPVHQRIVYTGKQLADDLTAKHYNLERGS--VLHLVLALRGGCC-------------
M.lychnidis(U5H805)            ---MNIKIKTLTGKEIDL-DIEPNDTVERIKVALEEKEGIPPAQQRLIFMGKAMPEEKTASDLKVTPGS--TLHLILSLRGGQC-------------
C.albicans(A0A1D8PJP6)         ---MQIKVKTLTGRDMPL-DVEPQDKIIRIKEMMEEKEGISPAQQRLIFNGSQLNDDQTVQEAGIQAGA--SLHLVLTLRGGN--------------
M.pulcherrima(A0A4P6XIP0)      ---MQVKVKTLTGRDIPV-DVEPTDKVIRIKEMMEEKEGIPPAQQRLIFNGSQLNDDATVEAAGIQPGT--SLHLVLTLRGGL--------------
R.necatrix(A0A1S7UJG8)         ---MMIKVRTLTGKLIEF-DVEPMDTIEDVKKRVEEKEGILPAQQRLIYSGKQMHDDVKLGEGGVVPDA--TLHLVLTLRGGASPRQW---------
S.cerevisiae(Q03919)           ---MIVKVKTLTGKEISV-ELKESDLVYHIKELLEEKEGIPPSQQRLIFQGKQIDDKLTVTDAHLVEGM--QLHLVLTLRGGN--------------
K.saulgeensis(A0A1X7R683)      ---MFIKVKTLTGKEIEV-ELQETDRIYHIKELLEQKEGIPPSQQRLIFQGKQINDEDSVGDAKLVDGM--QLHLVLTLRGGQ--------------
O.sativa(Q7XCL5)               MSMITVKVKTLTGKEVEV-SIEATETVARIKEQVEAAEGIPPPQQTLIYGGRQLADDMTAEMCDLRHGS--ELHLVLALRGGLL-------------
```

```
A.mphiamblys(A0A1J5WMY2)           ---MLIKVRTLTGAENEI-NIGADEKAEKIKRLVEDTDGIPMAQQRLLHNGRQVQDSKTLREIGVESGA--VLHLVIALRGG---------------
Nematocida(A0A177EH30)             ---MHIKIKTLEGTEVQL-EVDKAMKVKQVKELLQEKEAISIEQQRLIYGGKQLVDANTLETYGIQNND--VLHLVLALRGG---------------
P.knowlesi(A0A384LGS4)             ---MQILVKTLTGKRQSF-NFEPSSSVFQIKMAIEEKEGIDAKQIRLIYSGKQMHDDMKLSDYRVVPGS--TIHMILQLRGG---------------
P.vivax(A0A1G4HJQ8)                ---MQILVKTLTGKRQSF-NFEPSSSVFQIKMAIEEKEGIDAKQIRLIYSGKQMHDDMKLSDYRVVPGS--TIHMILQLRGG---------------
P.vivax_like(PVL_000121000)        ---MQILVKTLTGKRQSF-NFEPSSSVFQIKMAIEEKEGIDAKQIRLIYSGKQMHDDMKLSDYRVVPGS--TIHMILQLRGG---------------
P.inui(C922_00503)                 ---MQILVKTLTGKRQSF-NFEPSSSVFQIKMAIEEKEGIDAKQIRLIYSGKQMHDDMKLSDYRVVPGS--TIHMILQLRGG---------------
P.fragile(AK88_00191)              ---MQILVKTLTGKRQSF-NFEPSSSVFQIKMAIEEKEGIDAKQIRLIYSGKQMHDDMKLSDYRVVPGS--TIHMILQLRGG---------------
P.cynomolgi(PCYB_142240)           ---MQILVKTLTGKRQSF-NFEPSSSVFQIKMAIEEKEGIDAKQIRLIYSGKQMHDDMKLSDYRVVPGS--TIHMILQLRGG---------------
P.gallinaceum(A0A1J1GYI6)          ---MQILVKTLTGKRQSF-NFEPSSSVYQIKMAIEEKEGIDAKQIRLIYSGKQMHDDMKLSDYRVVPGS--TIHMILQLRGGII-------------
P.relictum(A0A1J1HAD2)             ---MQILVKTLTGKRQSF-NFEPSSSVYQIKMAIEEKEGIDAKQIRLIYSGKQMHDDMKLSDYRVVPGS--TIHMILQLRGGII-------------
P.falciparum(Q8IEI4)               ---MQILVKTLTGKRQSF-NFEPSSSVFQIKMAIEEREGIDAKQIRLIYSGKQMHDDMKLSDYRVVPGS--TVHMILQLRGG---------------
P.billcollinsi(PBILCG01_1313400)   ---MQILVKTLTGKRQSF-NFEPSSSVFQIKMAIEEREGIDAKQIRLIYSGKQMHDDMKLSDYRVVPGS--TVHMILQLRGG---------------
P.blacklocki(PBLACG01_1311100)     ---MQILVKTLTGKRQSF-NFEPSSSVFQIKMAIEEREGIDAKQIRLIYSGKQMHDDMKLSDYRVVPGS--TVHMILQLRGG---------------
P.reichenowi(PRCDC_1312000)        ---MQILVKTLTGKRQSF-NFEPSSSVFQIKMAIEEREGIDAKQIRLIYSGKQMHDDMKLSDYRVVPGS--TVHMILQLRGG---------------
P.praefalciparum(PPRFG01_1314900)  ---MQILVKTLTGKRQSF-NFEPSSSVFQIKMAIEEREGIDAKQIRLIYSGKQMHDDMKLSDYRVVPGS--TVHMILQLRGG---------------
P.adleri(PADL01_1311900)           ---MQILVKTLTGKRQSF-NFEPSSSVFQIKMAIEEREGIDAKQIRLIYSGKQMHDDMKLSDYRVVPGS--TVHMILQLRGG---------------
P.gaboni(PGABG01_1311100)          ---MQILVKTLTGKRQSF-NFEPSSSVLQIKMAIEEREGIDAKQIRLIYSGKQMHDDMKLSDYRVVPGS--TVHMILQLRGG---------------
P.ovale(A0A1C3L4R7)                ---MQILVKTLTGKRQSF-NFEPSSSVYQIKMAIEEREGIDAKQIRLIYSGKQMHDEMKLSDYRVVPGS--TIHMILQLRGGIF-------------
P.yoelii(A0A077YAC8)               ---MQILVKTLTGKRQSF-NFEPSSSVLQIKMAIEEREGIDAKQIRLIFSGKQMHDDMKLSDYRVIPGS--TIHMILQLRGGTI-------------
P.vinckei(YYE_01216)               ---MQILVKTLTGKRQSF-NFEPSSSVLQIKMAIEEREGIDAKQIRLIFSGKQMHDDMKLSDYRVIPGS--TIHMILQLRGGRI-------------
P.chabaudi(A0A077TQI6)             ---MQILVKTLTGKRQSF-NFEPSSSVLQIKMAIEEREGIDAKQIRLIFSGKQMHDDMKLSDYRVIPGS--TIHMILQLRGGRI-------------
P.berghei(A0A509AZB5)              ---MQILVKTLTGKRQSF-NFEPSSSVLQIKMAIEEREGIDAKQIRLIFSGKQMHDDMKLSDYRVIPGS--TIHMILQLRGGTN-------------
P.malariae(A0A1C3L273)             ---MQILVKTLTGKRQSF-NFEPSSTVYQIKMAIEEREGIDAKQIRLIYSGKQMHDEMKLSDYKVVPGS--TIHMILQLRGGKY-------------
T.gondii(A0A125YX02)               ---MQILIKTLTGRKQSF-NFEPDNTVLHVKQALQEKEGIDVKQIRLIYSGKQMSDELKLSDYKVVPGC--TIHMVLQLRGGC--------------
H.hammondi(HHA_254600)             ---MQILIKTLTGRKQSF-NFEPDNTVLHVKQALQEKEGIDVKQIRLIYSGKQMSDELKLSDYKVVPGC--TIHMVLQLRGGC--------------
N.caninum(F0V9J4)                  ---MQILIKTLTGRKQSF-NFEPDNTVLHVKQALQEKEGIDVKQIRLIYSGKQMSDELKLSDYKVVPGC--TIHMVLQLRGGC--------------
C.parvum(A3FPR3)                   ---MQILVKTLTGKKQNF-NFEPENTVLQVKQALQEKEGIDVKQIRLIYSGKQMSDDLRLLDYKVTAGC--TIHMVLQLRGGLR-------------
C.cayetanensis(A0A1D3D9Z9)         ---MQILVKTLTGRRQSF-NFEGENLVVHVKQALQEKEGVDVEQIRLIFSGKQMNDELKLSDYRVSPGC--TIHMVLQLRGGQSH------------
E.histolytica(C4LVJ5)              ---MLINIKLLNGRILSI-DLEPTDKISDLKAKLEEIEGITPEQQRLVFGGRQLGDDKTLQELNIQPGT--QINLLLALRGGF--------------
T.brucei(Q584D3)                   ---MLLKVKTVSNKVIQITSLTDDNTIAELKGKLEESEGIPGNMIRLVYQGKQLEDEKRLKDYQMSAGA--TFHMVVALRAGC--------------
Consensus                             M-IKVKTLTGKE----D-EP---V--IKE--EEKEGIPP-QQRLI--GKQM-D-KT--DY----GS---LHLVLALRGG
                                                                     **                                          *  ↑
```

Fig. S2. Sequence alignment and phylogenetic analysis of NEDD8 proteins. The NEDD8 protein sequences of *Plasmodium* species (grey shaded) were compared with 139 NEDD8 sequences of the indicated organisms representing metazoans, plants, fungi and protozoans. The ID of each NEDD8 protein is in the bracket. The conserved amino acid residues are in red font. The amino acid residues shared by at least 75% of the proteins in the alignment are in green font. The consensus sequence shared by at least 75% of the proteins in the alignment is shown in purple font, with hyphens representing variation at these positions. The C-terminal tail processing site in marked with an arrow and amino acid residues marked with asterisk have been demonstrated to confer specificity for neddylation enzymes. Amino acid residues in the blue font are conserved across *Plasmodium* NEDD8 proteins, but drastically differ from the consensus in physicochemical properties.

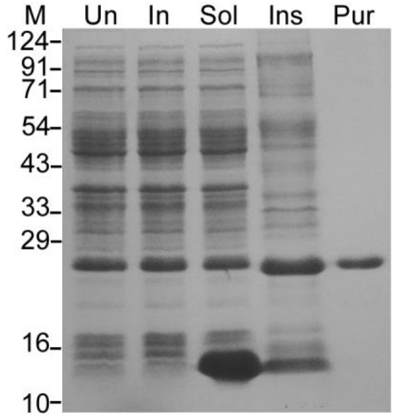

Fig. S3. Expression and purification of recombinant PfNEDD8. It was expressed as a C-terminal fusion of His-Sumo in *E. coli*. The His-Sumo-PfNEDD8 fusion protein (SN8) was purified by Ni-NTA chromatography under native conditions. The coomassie stained SDS-PAGE shows lysates of uninduced (Un) and induced (In) bacteria, soluble (Sol) and insoluble (Ins) fractions of the induced bacteria, and Ni-NTA purified SN8 (Pur). The protein size markers are in kDa (M).

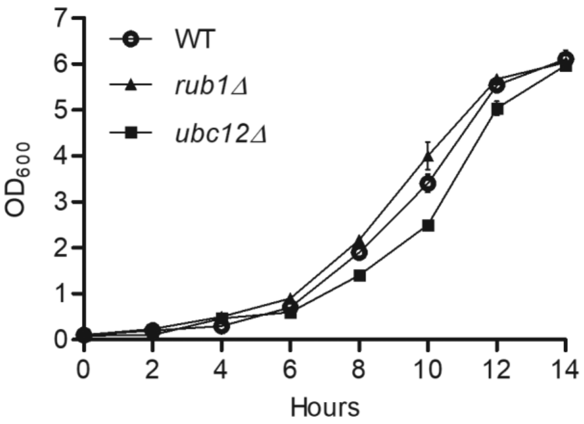

Fig. S4. Growth comparison of wild type, *rub1*Δ and *ubc12*Δ *S. cerevisiae* strains. The *rub1*Δ, *ubc12*Δ and wild type (WT) *S. cerevisiae* strains were grown in YPD medium for 14 hours beginning with 0.1 OD_{600}. Aliquots were collected at every two hours for the measurement of cell density at OD_{600}. The graph shows growth as OD_{600} on y-axis versus time on x-axis.

PfHN8-F	AATT**AGATCT**CAAAATGTATCCATATGATGTTCCAGATTACGCTGGAAGTCAA ATATTAGTTAAAACATTAACAGGG (HA-tag is underlined)
PfN8ex-R	ATT**CTCGAG**TTATCCTCCTCTTAATTG
PfN8ggM-R	AAT**CTCGAG**TTATGCTGCTCTTAATTG (codons for Gly75-Gly76 substitution to Ala-Ala are underlined)
SN8-F	AGAGAACAGATTGGAGGCATGCAAATATTAGTTAAAACATTAACAG
SN8-R	CTCGAGTGCGGCCGC**AAGCTT**TTATCCTCCTCTTAATTGTAAAATT
SMT3-F	ATTA**CATATG**CATCACCATCACCATCACTCGGACTCAGAAGTCAATCAA (His-tag sequence is underlined)
SMT3-R	AATC**AAGCTT**AAATACGTAGGCCTCCAATCTGTTCTCTGTGAGC
ScN8KO-F	CGACAGAGGAATAAATAAAGGAAGGTAATTAACTTCCTTACAGCCGTAACCG ATGCGTACGCTGCAGGTCGAC (sequence of ScRub1 locus is underlined)
ScN8KO-R	TCTTTTCTAATGAACACCTTCGATAAAATTCCATAAATGACGGAAAATGGTTC TAATCGATGAATTCGAGCTCG (sequence of ScRub1 locus is underlined)
ScN8-Fcon	CTTCGGGAAGGCAAACGAACAAC
ScN8-Rcon	GTAGCCTTCCAAAGTCCAAGTGAAC
ScU12KO-F	TACTTAGAGACATTAAAGAATCAGTTAGAGAATATAAAACAAGATAATAAAA ATGCGTACGCTGCAGGTCGAC (sequence of ScUbc12 locus is underlined)
ScU12KO-R	ATTTTATTGTTTATATATAATCAAATCAACCGAAAATTACTCTGAACTTGACTC AATCGATGAATTCGAGCTCG (sequence of ScUbc12 locus is underlined)
ScU12-Fcon	CAATCCTCTGGTAAATCTCATCT
ScU12-Rcon	ACTAACAGAAGATGGCGGTAGC
ScN8-Fepi	ACCGCCG**GAATTC**ATGATTGTTAAAGTGAAGACACTG
ScN8-Repi	ATGCGC**GGATCC**CTAGTTACCACCTCTTAGTGTTAATAC
PfN8-FScepi	AAAACG**GAATTC**ATGCAAATATTAGTTAAAACATTAACAG
PfN8-Rsp	TTAA**GGATCC**TTATCCTCCTCTTAATTGTAAAATC

Table S1. List of the primers used in the study. The sequences are in 5'-3' direction and restriction enzyme sites are in bold.

Data file S1. Compiled raw MS data of the pull-down of in vitro neddylation assay in the presence or absence of ATP. The +ATP is a test reaction and -ATP is a negative control reaction. Proteins exclusive to +ATP reaction are listed in Table 1 of the article.

Data file S2. Compiled raw MS data of the immunoprecipitates of *rub1*Δ[HA-PfN8] and wild type *S. cerevisiae* strains. Proteins exclusive to *rub1*Δ[HA-PfN8] are listed in Tables 2 and 4 of the article.

Data file S3. Compiled raw MS data of the immunoprecipitates of *rub1*Δ[HA-*rub1*] and wild type *S. cerevisiae* strains. Proteins exclusive to *rub1*Δ[HA-*rub1*] are listed in Tables 3 and 4 of the article.

Data file S4. Compiled raw MS data of the immunoprecipitates of HN8 and wild type *P. falciparum* parasites. Proteins exclusively to HN8 are listed in Table 5 of the article.

Extended size figures (these figures have larger size cropped images)

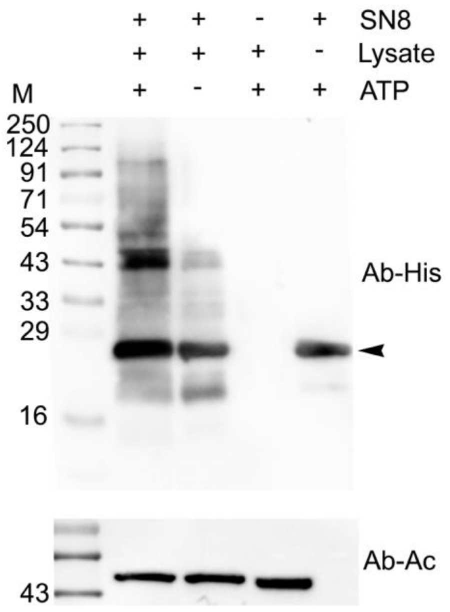

Fig. 2. Western blot analysis of in vitro neddylation. Conjugation of recombinant SUMO-PfNEDD8 (SN8) was carried out using parasite lysate and ATP at 37°C for 1 hour. The reactions were stopped and subjected to immunoblotting using anti-His antibody. The presence (+) or absence of reaction component is indicated. The arrow head indicates signal corresponding to unconjugated SN8. β-actin was used as a loading control for reactions containing the parasite lysate. The positions of proteins size markers are shown in kDa (M).

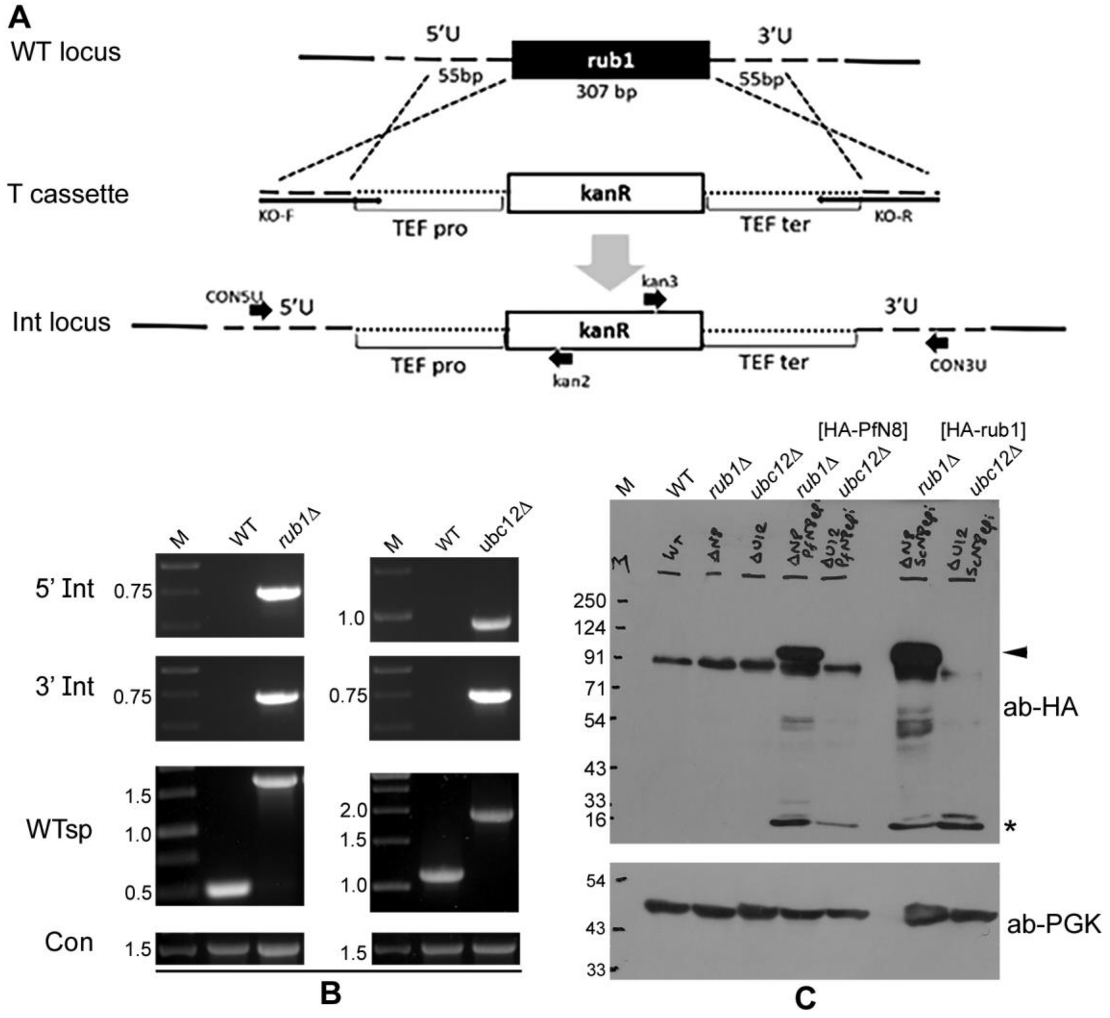

Fig. 3. Complementation of ScRub1 by PfNEDD8. A. Schematic for generation of *rub1*Δ or *ubc12*Δ. The wild type locus (WT locus) was replaced with a linear kanamycin cassette flanked by the homology regions of target locus (T cassette) via a double crossover homologous recombination, resulting in the integration locus (Int locus). The kanamycin coding sequence (kanR) is under the control of translation elongation factor promoter (TEF pro) and terminator (TEF ter). The horizontal arrows indicate positions of primers used in the analysis of knockout strains. **B. Confirmation of knockout.** The knockout was confirmed by PCR of the gDNAs of wild type (WT), *rub1*Δ and *ubc12*Δ strains using locus-specific primers (5' Int for 5' integration locus, 3' Int for 3' integration locus, WTsp for wild type locus and Con for ScAtg18). The ethidium bromide stained agarose gel shows PCR products, with DNA markers in kbp (M). **C. Western blot analysis of complemented strains.** HA-PfNEDD8 (HA-PfN8) or HA-ScRub1 (HA-rub1) were episomally expressed in *rub1*Δ and *ubc12*Δ strains. The lysates of wild type (WT), *rub1*Δ, *ubc12*Δ, HA-PfNEDD8-complemented *rub1*Δ or *ubc12*Δ (HA-PfN8), and HA-ScNEDD8-complemented *rub1*Δ or *ubc12*Δ (HA-rub1) strains were processed for western blotting using anti-HA antibodies (ab-HA). The lanes containing lysates of complemented strains show a free NEDD8/Rub1 protein (marked with an asterisk), whereas the lanes with *rub1*Δ[HA-PfN8] and *rub1*Δ[HA-*rub1*] strains also contain a high molecular weight band (marked with an arrow head) and some lower molecular weight conjugates, which are absent in other lanes. Phosphoglycerate kinase (anti-PGK) was used as a loading control. The protein size markers are in kDa (M).

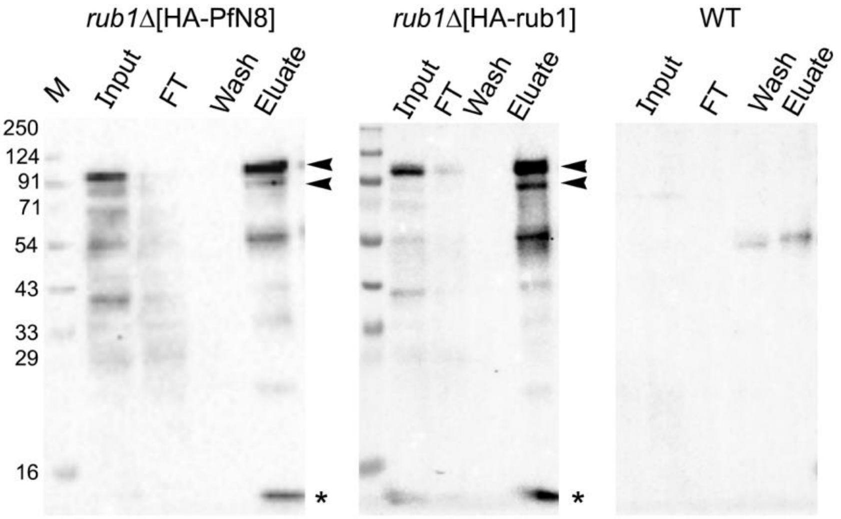

Fig. 4. Immunoprecipitation of PfNEDD8 and Rub1 from complemented strains. The lysates of *rub1*Δ[HA-PfN8], *rub1*Δ[HA-rub1] and wild type (WT) strains were immunoprecipitated with mouse anti-HA antibody. The eluates (Eluate) along with the extract (Input), flow through (FT) and wash (Wash) were analyzed by western blotting using rabbit anti-HA antibodies. The eluate lanes contain the respective target protein (indicated by an asterisk), and two prominent high molecular weight bands (indicated by arrow heads). The sizes of protein size markers are in kDa (M).

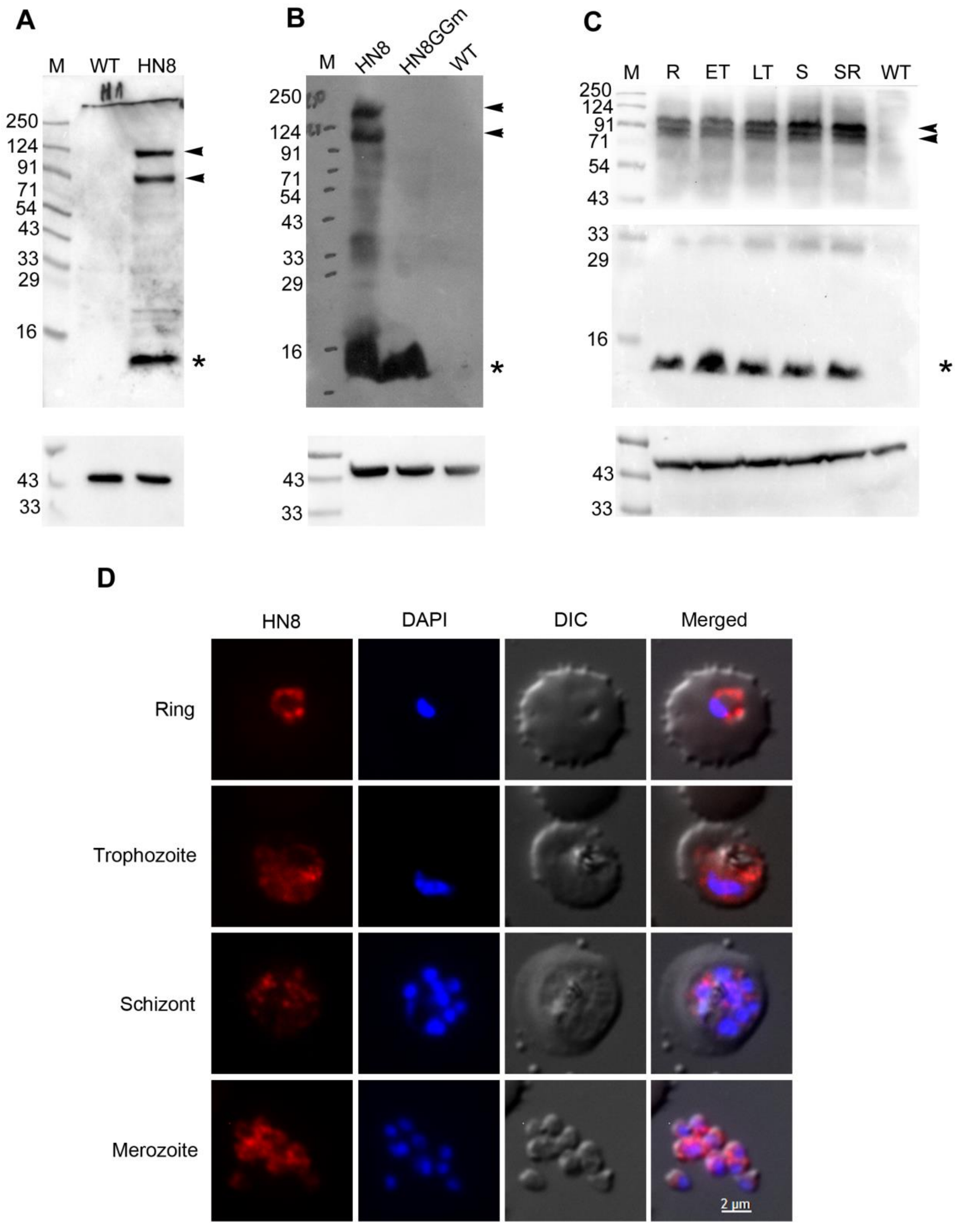

Fig. 5. Expression, conjugation and localization of *P. falciparum* NEDD8. A. Lysates of the wild type (WT) and HA-PfNEDD8–expressing (HN8) *P. falciparum* parasites were processed for western blotting using anti-HA antibodies (ab-HA). β-actin was used as a loading control (ab-Ac). The blot shows a prominent band close to the predicted size of HA-PfHANEDD8 (indicated with an asterisk) and two high molecular bands (indicated with arrow heads) in the HN8 lane only. **B.** The HA-PfNEDD8-expressing (HN8), mutant HA-PfNEDD8 (HN8GGm) and wild type (WT) parasites were processed for western blotting

using anti-HA antibodies (ab-HA), and β-actin was used as a loading control (ab-Ac). Note that the prominent high molecular bands (indicated with arrow heads) are present in HN8 lane only, whereas a band of the predicted size of HA-PfHANEDD8 or mutant HA-PfNEDD8 (indicated with an asterisk) is present in both HN8 and HN8GGm lanes. **C.** A synchronized culture of HA-PfNEDD8–expressing *P. falciparum* parasites was harvested at ring (R), early trophozoite (ET), late trophozoite (LT) and schizont/ring (SR) stages, and the parasites ($1{\times}10^8$ parasites/lane) were processed for western blotting using anti-HA antibodies (ab-HA). β-actin was used as a loading control (ab-Ac). The blot shows two prominent high molecular weight bands (indicated by arrow heads) along with the free HA-NEDD8 (indicated by an asterisk), indicating neddylation throughout the erythrocytic cycle. The double line in the top panel marks two different blots, which were developed separately to minimize over saturation of the blot due to the band around 12 kDa. The protein size markers in A, B and C are in kDa (M). **D.** Fixed HA-NEDD8-expressing *P. falciparum* parasites of the indicated stages were evaluated for localization of HA-NEDD8 using anti-HA antibodies. The images show HA-NEDD8 specific signal (HN8), nucleic acid staining (DAPI), the parasite and the erythrocyte boundaries (DIC), and the merged of all three images (Merged). The HA-NEDD8 signal is present throughout the parasite in all the stages shown, except the food vacuole. The scale bar shown is identical for all the images.

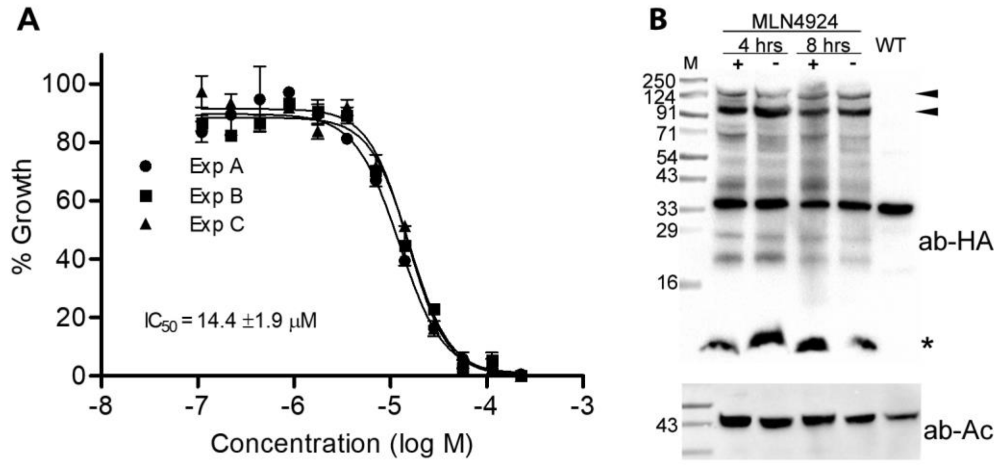

Fig. 6. The effect of MLN4924 on neddylation. A. Wild type *P. falciparum* D10 ring stage parasites were incubated with various concentrations of MLN4924 for 48-50 hours, and the % growth (y-axis) at different MLN4924 concentrations (x-axis) was plotted to determine 50% inhibitory concentration (IC_{50}) as described in the method section. The graph shows data from three independent experiments, with each point representing mean of two replicates. The IC_{50} value is average of three experiments with SD. **B.** The HA-PfNEDD8-expressing *P. falciparum* trophozoite stage parasites were grown with MLN4924 (+) or DMSO (-) for 4 or 8 hours, and then processed for western blotting using anti-HA antibodies (ab-HA). β-actin was used as a loading control (ab-Ac), and untreated wild type parasite lysate (WT) was used as another control for cross-reactivity of anti-HA antibodies. The wild type lane is not shown in Fig. 6 of the article. The blot shows multiple high molecular weight bands along a band of the predicted size of free HA-PfNEDD8 (indicated with an asterisk). The intensity of high molecular weight bands in MNL4924-treated parasites for both the time points appears to be comparable to that of DMSO-treated ones. The protein size markers are in kDa (M).

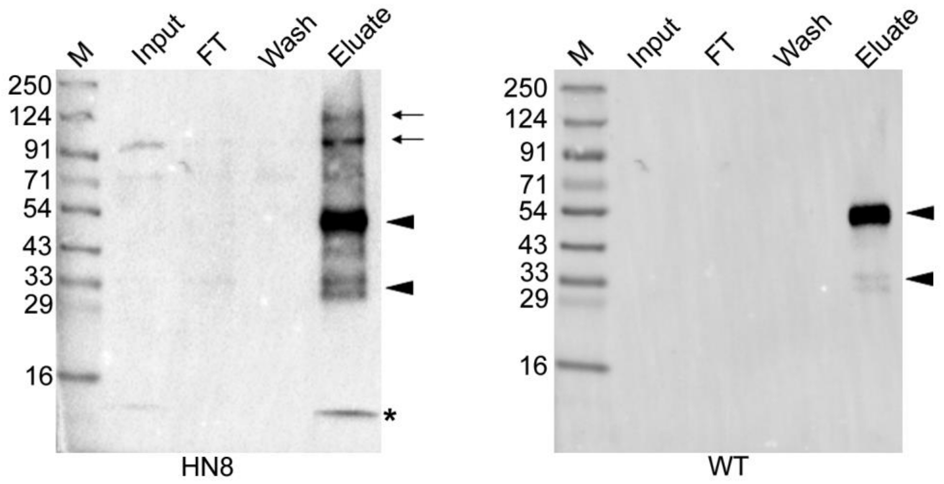

Fig. 7. Western blot of parasite immunoprecipitates. The lysates of HA-PfNEDD8-expressing (HN8) and wild type (WT) parasites were immunoprecipitated with rabbit anti-HA antibodies. The eluates (Eluate) along with the extract (Input), flow through (FT) and wash (Wash) were analyzed by western blotting using mouse anti-HA antibodies. The HN8 blot shows bands corresponding to free HA-PfNEDD8 (indicated with an asterisk) and high molecular weight conjugates (indicated with arrows). The arrow heads indicate non-specific signal in both the blots. The protein size markers are in kDa (M).
